# Cerebrospinal Fluid Levels of AFP and hCG: Validation of the Analytical Method and Application in the Diagnosis of Central Nervous System Germ Cell Tumors

**DOI:** 10.3390/diagnostics11111980

**Published:** 2021-10-26

**Authors:** Annamaria D’Alessandro, Domenico Ciavardelli, Anna Pastore, Germana Giannone, Giada Del Baldo, Andrea Carai, Angela Mastronuzzi, Andrea Onetti Muda, Ottavia Porzio

**Affiliations:** 1 Clinical Biochemistry Laboratory, IRCCS “Bambino Gesù” Children’s Hospital, 00165 Rome, Italy; germana.giannone@opbg.net (G.G.); ottavia.porzio@opbg.net (O.P.); 2 School of Medicine and Surgery, University “Kore” of Enna, 94100 Enna, Italy; domenico.ciavardelli@unikore.it; 3 Center for Advanced Studies and Technology (C.A.S.T.), University “G. d’Annunzio” of Chieti-Pescara, 66100 Chieti, Italy; 4 Research Unit of Diagnostical and Management Innovations, IRCCS “Bambino Gesù” Children’s Hospital, 00165 Rome, Italy; andrea.onettimuda@opbg.net; 5 Department of Onco-Hematology, Cell Therapy, Gene Therapy and Hemopoietic Transplant, IRCCS “Bambino Gesù” Children’s Hospital, 00165 Rome, Italy; giada.delbaldo@opbg.net (G.D.B.); angela.mastronuzzi@opbg.net (A.M.); 6 Department of Neurosciences, IRCCS “Bambino Gesù” Children’s Hospital, 00165 Rome, Italy; andrea.carai@opbg.net; 7 Department of Experimental Medicine, University of Rome “Tor Vergata”, 00133 Rome, Italy

**Keywords:** AFP, hCG, cerebrospinal fluid, germ cell tumors, central nervous system tumors

## Abstract

The determination of Human Chorionic Gonadotropin (hCG) and Alpha Fetoprotein (AFP) levels on serum and amniotic fluid plays a fundamental role in the diagnosis and follow-up of specific physiological or pathological conditions (e.g., pregnancy, threat of abortion or germ cell tumors). Recently, the quantification of hCG and AFP in other biological fluids has gained great attention to support the diagnosis, prognosis and follow-up of neoplastic diseases deriving from trophoblastic cells, such as germinomas. Most of the commercial kits for hCG and AFP assays are developed to be used on biological fluids such as serum/plasma and/or urine by manufacturing companies. The aim of this work was to evaluate the suitability of the analytical method certified for the use on serum, and/or amniotic fluid for the quantification of hCG and AFP in cerebrospinal fluid, carrying out an internal validation protocol. The data reported here show that the automated immunochemical method is fit for quantification of hCG and AFP in cerebrospinal fluid (CSF), allowing selective and specific diagnosis of secreting germ cell tumors. This is confirmed by the positive correlation between elevated levels of hCG or AFP and the diagnosis of brain tumors.

## 1. Introduction

The determination of the levels of alpha-fetoprotein (AFP) and Human Chorionic Gonadotropin (hCG) on serum and amniotic fluid plays a fundamental role in the diagnosis, prognosis and follow-up of specific physiological or pathological conditions such as pregnancy, threat of abortion and germ cell tumors (GCTs). Recently, there has been great interest in the possibility of determining the values of hCG and AFP in other organic fluids such as cerebrospinal (CSF) [1,2].

GCTs are a heterogeneous group of cancers in terms of age of onset, location, clinical presentation and prognosis, and represent 2–3% of pediatric malignancies [3].

Germ cell tumors (GCTs) of the central nervous system (CNS) are classified according to the World Health Organization (WHO) into pure germinomas and non-germinomatous germ cell tumors (NGGCTs) [4]. The presence of specific protein markers produced by germ cell tumors has been extremely important in helping to diagnose GCTs. AFP is detected in endodermal sinus tumors such as yolk sac tumors, and β-HCG is a marker of choriocarcinomas [5].

When the suspicion of an intracranial GCT arises, the increase of serum or CSF markers could precede the radiological finding by several months, resulting in a high predictive/diagnostic power [6]. The relative increase in hCG and AFP values at the serum and/or CSF level is also of fundamental importance not only for diagnosis, but also for monitoring the response to treatments and follow-up, even in the absence of histological data [6]. The persistence of high values of these markers after surgery or its increase after the resolution of disease also represents an important index of persistence/resurgence of the disease [6].

Most of the commercial kits for hCG and AFP assays are developed to be used on biological fluids such as serum and/or amniotic liquid by manufacturing companies. The aim of this work was to evaluate whether the analytical method already used in the Clinical Biochemistry Laboratory of the Bambino Gesù Children’s Hospital for the determination of hCG, an AFP on established biological fluids, could also be used on CSF. As matrix effects can influence test results when alternative sample types are used, and alternative sample types should always be validated before clinical use, we carried out the CSF assay of both AFP and hCG validation using an internal validation protocol according to the Eurachem Guide [7].

## 2. Materials and Methods

### 2.1. Experimental

#### 2.1.1. Chemicals and Instrumentation

We used the ADVIA Centaur^®^ XPT Immunoassay System (Siemens Healthineers Diagnostics, Erlangen, Germany) for AFP and hCG determinations in CSF samples and serum. The diagnostic kits used in this study to determine the concentrations of AFP and hCG were a kind gift from the Siemens Healthineers Diagnostics (Erlangen, Germany). The AFP and hCG assays are a two-site sandwich immunoassay based on direct chemiluminometric technology, which uses constant amounts of two antibodies. The first antibody for the AFP assay is a rabbit polyclonal anti-AFP affinity purified antibody labeled with acridinium ester, and the second is a mouse monoclonal anti-AFP antibody covalently conjugated to paramagnetic particles. For hCG assay, the first antibody is an anti-hCG goat polyclonal purified and labeled with acridinium ester, and the second is a purified mouse monoclonal anti-hCG antibody, conjugated covalently to paramagnetic particles. These two antibodies are specific for different epitopes present on the free ß-subunit and on the ß-subunit of the intact hCG.

#### 2.1.2. CSF Samples

The validation study was performed using 370 leftover CSF samples sent to the Clinical Biochemistry Laboratory of the Bambino Gesù Children’s Hospital for physical and chemical evaluation. In particular, the CFS samples used in this study were selected based on medical records showing no documented history of blood–brain breakdown or CNS infection. Clear and colorless CSF samples with a leukocyte count ≤5 and normal values of proteins, glucose and chloride, and levels of AFP and hCG comparable with those found in HPLC-grade water (0.90 ng/mL and 2.78 mIU/mL, respectively) were pooled and used as blank matrix. 

#### 2.1.3. Study Population

We collected the leftover CSF samples obtained from 41 patients aged from 3 months to 20 years presented to the Bambino Gesù Children’s Hospital with radiological diagnosis of brain lesion suspected for GCT, and we analyze both hCG and AFP. We performed hCG and AFP assays on the serum from the same patients before and after the CSF collection, in order to compare the results for diagnostic and prognostic–therapeutic purposes. In addition, the medical records of each patient were examined for the evaluation of the cytological/histological or radiological data that could be useful for patient diagnosis and follow-up.

### 2.2. Method Validation

hCG and AFP concentration were measured using ADVIA Centaur^®^ XPT Immunoassay System, previously validated only for serum and/or amniotic fluid specimens. In this study, we validated its use for CSF samples. CSF hCG and AFP assays were validated through evaluation of linearity [8], limit of detection (LOD), limit of quantification (LOQ), repeatability, and trueness [7].

#### 2.2.1. Linearity

Linearity was studied according to IUPAC guidelines [8]. One instrumental blank and five or eight calibration standards for AFP and hCG, respectively, were analysed to calculate the calibration functions. Three experimental replicates were run for each standard. Homoscedasticity was verified by applying Bartlett’s tests. When the assumption of homoscedasticity of the responses was violated, the weighted least squares fitting was applied. The best weighting factor was selected between 1/X and 1/X2 (X = concentration of the calibration standard) using the variance test [9,10], evaluating the percentage relative error (RE%) calculated with the following formula:

RE% = [(Xf − Xex)/Xex] × 100(1)

where Xf is the concentration computed from the regression equation obtained using each weighting scheme and Xex is the nominal standard concentration [11] and checking for linearity using Mandel’s fitting test.

#### 2.2.2. Analytical Performance

Limit of detection (LOD) and limit of quantification (LOQ) were calculated by analysing ten experimental blanks. LOD and LOQ were calculated with the following formula:

YLOD = Yb + 2t SDb(2)

YLOQ = Yb + 10 SDb(3)

where SDb = standard deviation of the mean blank signal, and t is the constant from one-sided Student’s *t*-test (95% confidence level) for *n* − 1 degree of freedom [8,12]. The corresponding concentrations were calculated using the weighted calibration functions.

For upper-end sensitivity studies, pooled CSF samples were spiked by serially diluted serum samples containing high amounts of AFP, obtaining CSF concentrations of 15, 45, and 125 ng/mL for AFP and 35, 125, and 600 mIU/mL for hCG.

Precision was calculated in terms of repeatability (intraday precision) as relative standard deviation (RSD%) [8].

Trueness was assessed from the recovery assay. In order to verify possible matrix effects as constant and proportional systematic errors, the recovery function over the entire working range was calculated [12]. Recovery function was obtained by calculating the concentration (Xf) of matrix-matched calibration levels from the corresponding signals (Yf) when using the external calibration fit, with the following formula:

Xf = (Yf − b0ex)/b1ex(4)

and plotting the values obtained versus the external calibration concentrations (Xex):

Xf = b0f + b1fXex(5)

When both constant and proportional systematic errors are negligible, the calculated intercept (b0f) and slope (b1f) of recovery function should not significantly differ from 0 and 1, respectively, at a defined confidence level.

#### 2.2.3. Statistical Analysis

The homogeneity of the variance was evaluated using the Levene test. The significance of the differences for CSF levels of AFP and hCG among the study groups was assessed by one way analysis of the variance (ANOVA) followed by Fisher’s least significant difference (LSD) post hoc test or Kruskal–Wallis test followed by multiple comparison of mean ranks. After mean centering, unit variance scaling, and log transformation of AFP and hCG concentrations, unsupervised hierarchical cluster analysis and supervised multivariate partial least squares-discriminant analysis (PLS-DA) were performed to explore possible clustering of the subjects based on CSF levels of AFP and hCG. Receiver operating characteristic (ROC) analysis and the calculation of the area under curve (AUC) were performed to assess the sensitivity and the specificity of AFP and hCG levels for the diagnosis of NGGCT. Statistical analysis was performed using Statistica 6.0 (StatSoft, Tulsa, OK, USA) and MetaboAnalyst 5.0 module [13].

## 3. Results

### 3.1. Method Validation

Table 1 shows the analytical performance of the developed method. As the analytical responses did not meet the assumption of homoscedasticity for both AFP and hCG (Bartlett test, *p* < 0.050, 95% confidence level), weighted least squares fitting was applied to calculate the calibration functions over the investigated concentration ranges. We used the following weighting factors: 1/X and 1/X2 where X is the concentration of the calibration standard. For both AFP and hCG, the variance test scores indicated that the weight 1/X2 gives the smallest spread of the weighted variances compared with 1/X (data not shown). Accordingly, the calculated RE percentage was lower when 1/X2 is used for both analytes compared with 1/X (data not shown). However, the weighted calibration functions calculated weighting for 1/X2 showed significant deviation from linearity for both AFP and hCG at 95% confidence level (Mandel test, *p* = 0.001 and 0.025, respectively). Therefore, 1/X was chosen as the weighting factor. As shown in Table 1, the calculated LOD and LOQ indicate that the method provides suitable sensitivity to detect and quantify AFP and hCG in CSF. Furthermore, excellent repeatability (inter-day precision) was calculated and the relative standard deviations of Xf were even lower than 5% over the investigated calibration range for both analytes (Table 1). The trueness assessed from the recovery assay indicated that, over the investigate concentration ranges, R percentage was acceptable, ranging from 88% and 95% for AFP and 90% to 99% for hCG (Table 1). The intercept values of the calculated recovery functions did not significantly differ from the ideal value of 0, indicating a negligible constant systematic error for both analytes. In contrast, the values of the slopes of the recovery functions were found to be significantly different from the ideal value of 1, indicating that quantification of AFP and hCG in CSF is affected by proportional systematic errors due to the matrix effect. However, the values of the calculated slopes (0.91 and 0.971 for AFP and hCG, respectively) indicated that the deviation from the ideal value of 1 is always lower than 10%, suggesting that the method provides adequate accuracy for quantification of AFP and hCG in CSF.

### 3.2. Clinical Validation

**Figure 1:**
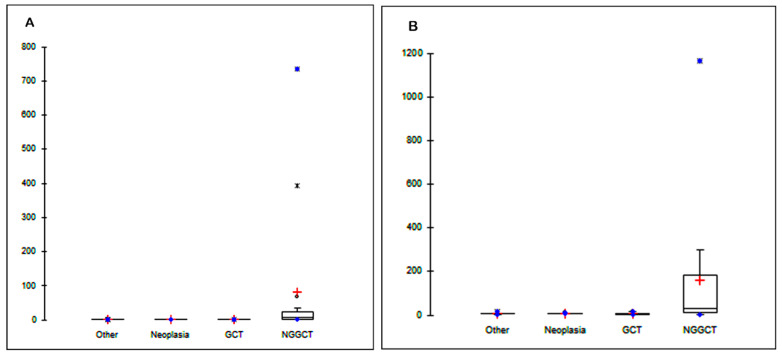
Cerebrospinal fluid (CSF) level of alpha-fetoprotein (AFP) (**A**) and human chorionic gonadotropin (hCG) (**B**) in patients affected by non-cancerous diseases (Other), other brain tumors (Neoplasia), germinoma (GCT), and non-germinomatous germ cells tumors (NGGCT). Dot plots show mean CSF concentrations (ng/mL) of AFP and hCG. * indicates *p*-values < 0.050 (Kruskal–Wallis test followed by multiple comparison of mean ranks).

The assumption of homoscedasticity of data was rejected by the Levene test (*p* < 0.001 for both AFP and hCG). Therefore, the Kruskal–Wallis test followed by multiple comparison of mean ranks was applied to evaluate the significance in the differences of AFP and hCG CSF levels between the study groups. Figure 1A shows the CSF levels of AFP. NGGCT patients show significantly higher values of AFP (mean ± SEM = 81 ± 50 ng/mL, SEM: standard error of the mean, *n* = 16) when compared to the other GCT patients (1.28 ± 0.02 ng/mL, *n* = 18, *p* = 0.014) and patients affected by other cancer types (1.300 ± 0.001 ng/mL, *n* = 20, *p* = 0.022). Furthermore, we found significantly higher levels of CSF hCG in NGGCT patients (158 ± 72 mIU/mL, *n* = 16) when compared with the other study groups (GCT, 6 ± 1 mIU/mL, *n* = 18, *p* < 0.001; Neoplasia, 7.2 ± 0.3 mIU/mL, *n* = 20, *p* < 0.001; Other, 7 ± 1 mIU/mL, *n* = 8, *p* = 0.004; Figure 1B).

**Figure 2:**
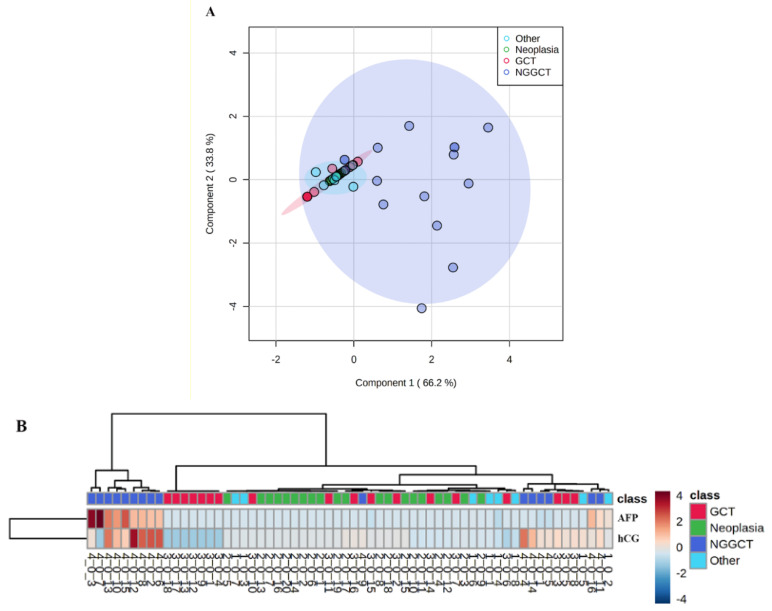
Supervised partial least square discriminants analysis and unsupervised hierarchical cluster analysis of cerebrospinal fluid (CSF) levels of alpha-fetoprotein (AFP) and human chorionic gonadotropin (hCG) in patients affected by non-germinomatous germ cells tumors (NGGCT), germinoma (GCT), other brain tumors (Neoplasia), and other diseases (Others). (**A**): PLS-DA score plot based on CSF concentrations of AFP and hCG found in the four study groups. (**B**): heatmap visualization of the hierarchical cluster analysis performed using the Euclidean distance. The bars represent the CSF levels of AFP and hCG with pseudo-color coding ranging from the blue (low level) to the red (high level) in accordance with AFP and hCG concentrations. The rows indicate the analysed samples.

**Figure 3:**
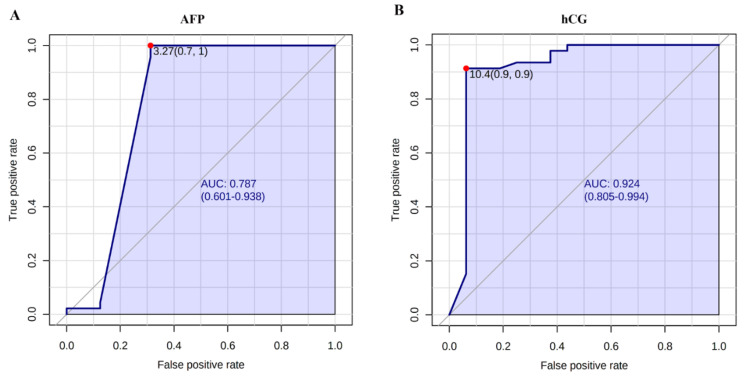
Receiver operating characteristic (ROC) analysis of cerebrospinal fluid (CSF) levels of (**A**): alpha-fetoprotein (AFP) and (**B**): human chorionic gonadotropin (hCG) in patients affected by non-germinomatous germ cells tumors (NGGCT) and subjects belonging to the other study groups. ROC analysis was performed in order to discriminate patients affected by NGGCT from the other study groups using CSF levels of AFP and hCG. The values of the area under the curve (AUC) with confidence intervals (95% confidence level) are shown along with optimal cut-off concentration values corresponding to higher specificity and sensitivity.

Based on CSF levels of AFP and hCG, supervised PLS-DA (Figure 2A) and unsupervised cluster analysis (Figure 2B) showed that NGGCT patients are grouped apart from GGCT patients and subjects affected by other brain tumors or other diseases such as diabetes. To assess the sensitivity and the specificity of CSF levels of AFP and hCG, ROC analysis was performed. The AUC value indicates the test strength, and the AUC of an ideal biomarker should be 1. AUC values lower than 0.7 indicate poor classification accuracy. In our study, ROC analysis resulted in an AUC of 0.787 and a cut-off value of 3.27 ng/mL of AFP provides sensitivity of 100% and specificity of 70%, (Figure 3A). The calculated AUC for hCG was 0.924 and the cut-off hCG concentration of 10.4 mIU/mL provides both sensitivity and specificity of 90% (Figure 3B), indicating that hCG was able to correctly identify the subject not affected by NGGCT and has a high positive predictive value.

## 4. Discussion

In this study, we first validated the Siemens Advia^®^ Centaur hCG and AFP methods for their use on CSF. Imprecision ranged from 1 to 12% for both assays. The recoveries were 88% and 95% for AFP and 90% to 99% for hCG, demonstrating a slight matrix effect. Our results are according to those reported by Mitsios and collaborators, showing an imprecision of about 10.2% and a recovery ranging from 113 to 129% [14]. Taken together, these results demonstrate that the use of the Siemens ADVIA^®^ centaur system for both AFP and hCG assay in CSF samples could be successfully performed.

GCTs are a heterogeneous group of cancers in regard to age of onset, location, clinical presentation and prognosis. They represent 2–3% of pediatric malignancies [3].

Germ cell tumors (GCTs) of the central nervous system (CNS) are classified according to the World Health Organization (WHO) into pure germinomas and non-germinomatous germ cell tumors (NGGCTs) [4]. CNS germ cell tumors are found mainly in the midline brain regions. The pineal gland and suprasellar region are the most frequent localizations but they occur also intraventricularly, in cerebral hemispheres, the medulla, thalamus and basal ganglia [14,15]. About 15% of pure germinomas occur simultaneously in pineal and suprasellar regions. NGGCTs are a group of neoplasms mostly located in pineal and/or suprasellar regions. They include yolk sac tumor (YST), embryonal carcinoma (EC), choriocarcinoma (CHC), and mixed malignant tumors in various combinations, often together with teratoma or germinoma components [15]. More than 90% of CNS GCTs arise in patients younger than 20 years with an incidence peak between 10 and 16 years [14,15,16]. Germinomas have a favorable prognosis with an overall survival greater than 90%, while for NGGCTs the survival rates is low, ranging from 40% to 70% [15]. Evidence of tumor spread outside the primary site has been reported in above 30% of cases [17].

The onset symptomatology of GCT is related to the localization of the tumor itself (i.e., intracranial hypertension in patients suffering from tumors germinomas originating from the pineal gland; diabetes insipidus and/or other endocrinological dysfunction, bilateral temporal hemianopsia in cases of involvement of the hypothalamic-pituitary area) [6]. Sometimes, the symptoms with involvement of the endocrine system can precede the alterations found in the radiological findings by several months. On Computed Tomography (CT) scan, germinomas are homogeneous, isodense to hyperdense and frequently associated with calcification. In Magnetic Resonance Imaging (MRI), germinoma is isointense on T1-weighted images and hypo-isointense on T2 [18]. On the other hand, NGGCTs are heterogenous on MRI and invasive than germinoma with a high frequency of cysts and hemorrhage [18]. In addition to a brain imaging evaluation, spine MRI and a CSF cytology examination must be performed to complete disease staging

Despite neuroradiological characteristics, in the absence of a clear bifocal localization and histological confirmation, diagnosis can be difficult and the assessment of both hCG and AFP CSF values would be fundamental. The presence of hCG and AFP in serum and/or CSF at levels above the maximum attended for age is diagnostic for NGGCTs and biopsy can be avoided. Yolk sac tumors can secrete AFP and choriocarcinomas can release hCG. Moreover, elevated AFP and hCG can sometimes occur with immature teratomas. Germinomas may be associated with mild increases in total hCG and may show elevated placental alkaline phosphatase (PLAP) [15]. AFP levels greater than 1000 ng/mL are considered high risk and may require intensified chemotherapy and radiation [19].

Differentiating pure germinomas from NGGCTs is crucial because treatments and prognoses are quite different. Without a positive tumor marker result, a biopsy is required for a definitive diagnosis [20].

The relative increase in hCG and AFP values at the serum and/or CSF level are also of fundamental importance not only for the diagnosis, but also for monitoring the response to treatments and the follow-up, even in the absence of histological data [6]. This was clearly demonstrated in the GETUG 13 phase III trial, which reports personalized chemotherapy based on the changes in AFP and hCG levels in poor-prognosis germ-cell tumors [21].

## 5. Conclusions

In our observational study aimed at evaluating the predictive value of AFP and hCG, we demonstrated that AFP provides a sensitivity and specificity of 90%, suggesting this biomarker is suitable for the diagnosis of NGGCT. Regarding hCG, we found a sensitivity of 50% and a specificity of 100%, indicating that this marker was able to correctly identify the subject not affected by NGGCT. The combination of the two biomarkers in a multivariate discriminant model could provide better sensitivity and specificity compared with that obtained using AFP or hCG alone, thus demonstrating how the combined use of hCG and AFP can represent valid support in the diagnosis and follow-up of NGGCT neoplastic diseases.

The determination of markers in non-standard samples represents, from an analytical point of view, a critical issue for the laboratory and requires a complex process of validation of the analytical method in order to certify its suitability. The results should be evaluated together with the patient’s clinical history and other diagnostic tools to obtain the most appropriate diagnostic framework.

## Figures and Tables

**Table 1:** Linearity of the weighted calibration functions, limit of detection (LOD), limit of quantitation (LOQ), repeatability (intra-day precision) and trueness of analysis of alpha-fetoprotein (AFP) human chorionic gonadotropin (hCG) in cerebrospinal fluid. The weighting factor 1/X was chosen between two weighting factors, i.e.,: 1/X and1/X2, based on the variance and Mandel test for both AFP and hCG. Accuracy was evaluated under repeatability conditions from analysis of spiked blank CSF. The expected spike concentrations and the corresponding recovery values are shown for three representative spike levels (low, medium, and high concentration level) of the six spikes that were analysed to calculate the recovery function.

	Calibration Function									Precision	Trueness	Recovery Function	
Analyte	Linear Range ^a^	Bartlett Test, *p* ^b^	b0 (CI) ^c^	b1 (CI) ^c^	*r^2^*	Mandel Test, *p* ^b^	LOD ^a^	LOQ ^a^	Spike Level ^a^	Measured Concentration, Mean ^a^ (SD ^d^, RSD% ^e^, *n* = 6)	R% ^f^ (SD ^d^, *n* = 6)	b0 (CI) ^c^	b1 (CI) ^c^
AFP	4.35–286	<0.001	2110 (197)	399 (20)	0.993	0.23	1	2	15	14.2 (0.5, 3.8)	95 (4)	0.2 (1.0)	0.91 (0.03)
									45	41 (2, 4)	91 (4)		
									125	117 (2, 3)	88 (6)		
hCG	4.59–695	<0.001	5471 (366)	846 (27)	0.994	0.27	3	4	35	34.5 (0.8, 2.4)	99 (2)	−2 (4)	0.971 (0.007)
									125	112 (1, 1)	90 (1)		
									600	570 (15, 3)	95 (7)		

^a^ Expressed in ng/mL for AFP and mIU/mL for hCG, respectively; ^b^ *p* values, 95% confidence level; ^c^ Confidence interval, 95% confidence level; ^d^ Standard deviation; ^e^ Relative standard deviation (%); ^f^ Recovery calculated as [(Xi − X0)/(XSi)] × 100 where Xi = concentration of the ith spiked sample, X0 = concentration of the unspiked sample, and XSi = known spike concentration (expressed in ng/mL for AFP and mIU/mL for hCG, respectively).

## Data Availability

The data that support the findings of this study are available from the corresponding author, [A.D.], upon reasonable request.
